# Peanut allergen characterization and allergenicity throughout development

**DOI:** 10.3389/falgy.2024.1395834

**Published:** 2024-08-27

**Authors:** Casey G. Cohen, Yael Levy, Diana Toscano-Rivero, Ekaterina Manasherova, Nancy Agmon-Levin, Ron S. Kenett, Bertrand J. Jean-Claude, Bruce D. Mazer, Ran Hovav, Mona I. Kidon

**Affiliations:** ^1^ Department of Pediatrics and Division of Experimental Medicine, McGill University and The Research Institute of The McGill University Health Centre (RI-MUHC), Montreal, Canada; ^2^ Plant Sciences Institute, Agricultural Research Organization, Volcani Center, Rishon LeZion, Israel; ^3^ Clinical Immunology, Angioedema and Allergy Unit, Center for Autoimmune Diseases, Sheba Medical Center, Tel Hashomer, Israel; ^4^ Safra Children's Hospital, Sheba Medical Centre, Tel Hashomer, Israel; ^5^ Samuel Neaman Institute, Technion, Haifa, Israel; ^6^ Department of Medicine and Division of Experimental Medicine, McGill University and Research Institute of The McGill University Health Centre (RI-MUHC), Montreal, Canada; ^7^ Sackler School of Medicine, Tel Aviv University, Tel Aviv-Yafo, Israel

**Keywords:** peanut, food allergy, seed development, storage proteins, skin prick test (SPT)

## Abstract

**Introduction:**

Peanut allergy (PA) in children is a major concern. There is a need for better biological material for both diagnosis and oral immunotherapy (OIT) treatments. The unique state of seeds at early reproductive stages may affect the allergenicity of storage proteins, and impact clinical diagnostic and OIT protocols. The objective of this study was to evaluate the major allergen content in sequential seed developmental stages and monitor allergenicity via specific IgE binding quantification and skin prick testing.

**Methods:**

Seeds were collected from peanut plants and sorted into five developmental stages: initial (S1), developing (S2), full-size without coloration (S3), full-size with coloration (S4), and fully mature (S5) seeds. Samples were characterized by RNA-Seq, ELISA, and immunohistochemistry. Lyophilized, ground preparations were used for evaluation of skin test responses in sixty challenge-proven PA children.

**Results:**

Gene expression, protein content, and specific IgE binding of allergenic proteins increased throughout seed maturation and development. An expression bias towards the less allergenic A-genome copy of the major allergen Ara h 2 was found in earlier stages, especially in stage S2. Immunohistochemical staining showed that Ara h 2 is more dispersed in the cell and less accumulated within organized bodies at stage S2 versus stage S4. Significant differences were found in mean wheal responses between the commercial peanut extract (equivalent to stage S5) and stages S1 and S2, but not with stage S4, upon skin prick testing in subjects with PA.

**Discussion:**

The observed decrease in peanut-specific IgE binding of immature peanut seeds may be a result not only of decreased amounts of allergenic proteins, but also of profound changes in seed composition and conformation. This may be significant for developing a safer and more effective peanut OIT protocol.

## 1 Introduction

Food allergy has emerged as a major health concern in the 21st century. Known as the “second wave” of the allergy epidemic, food allergy is a specific immune response to a particular food that can reproducibly cause adverse health effects upon exposure (1). Recent studies suggest that in Western countries, the prevalence of clinical food allergy among preschool children may be as high as 10% (2, 3).

Over the past recent decades, the incidence of peanut allergy has increased, affecting 1.5%–3% of children worldwide, making it a significant global public health problem (4, 5). Peanut allergy is particularly dangerous, with accidental exposure leading to life-threatening reactions and even fatalities. Unfortunately, peanut allergy does not spontaneously resolve in most children, and it is usually a lifelong condition with no known cure (6, 7). The current management approach is strict avoidance of the food and the constant availability of an epinephrine auto-injection in the case of accidental ingestion. In January of 2020, the US FDA approved Palforzia™, the first ever FDA-approved treatment for peanut allergy. This oral medication consists of capsules containing peanut allergen powders at precise doses and incrementally larger doses are to be ingested daily by allergic individuals, desensitizing them over time (8, 9). Despite the positive advancements reaching FDA approval, individuals still experience frequent allergic reactions and long-term daily compliance is not always feasible. Peanut allergy severely impacts quality of life of affected individuals and their families, causing heightened anxiety and limiting their participation in school and social events (10).

Several peanut proteins have been identified as allergens in various individuals and populations, titled Ara h 1 through Ara h 18 by the WHO/IUIS Allergen Nomenclature Subcommittee (11, 12). The cupin superfamily includes Ara h 1 and Ara h 3, while the prolamin superfamily includes the conglutins Ara h 2, Ara h 6, and Ara h 7, and are responsible for the majority of life-threatening reactions to peanuts. The non-specific lipid transfer protein, Ara h 9, the profilin, Ara h 5, and the birch pollen protein Bet v 1 homolog, Ara h 8, can cause allergic reactions that may be related to inhalation allergy to pollen due to the similarity in sequential and/or conformational molecules (13–15). The relatively large number of allergenic peanut proteins presents a challenge for the scientific and medical communities in discovering solutions to this serious problem.

Most seed-based allergies, including peanuts, are based on immune-mediated reactions to storage proteins, which are relatively stable to heat denaturation (16). In some cases, such as dry roasting of peanuts, there is evidence to suggest the allergenicity of the major allergens is enhanced (17, 18). Boiling can reduce the solubility of major peanut allergens and result in their loss into the cooking water, but it does not produce hypoallergenic material from peanuts (19, 20). The stability of peanut allergens to heat denaturation is at least partly due to the state of these storage proteins in the mature seed, where they accumulate in large and rigid protein bodies of about 5–10 μm in diameter (21). These organelles are bound by a single membrane and retain a highly stable homogeneous matrix in which crystalloids and/or globoids may be embedded. Members of these storage proteins, such as Ara h 1 and Ara h 2, are responsible for immediate, life-threatening reactions in peanut-allergic individuals with specific IgE for these allergens (22, 23).

The peanut seed undergoes several changes during its developmental process. Upon anthesis or flowering, the pod, which is a simple fruit structure that originates from a carpel, grows quickly and develops a large and juicy shell wall or pericarp (24). Initially, the pericarp occupies most of the fruit volume and serves as a temporary source of nutrients that are transferred to the nascent seed. As development progresses, the seed accumulates more nutrients and grows rapidly, eventually constituting most of the total pod volume at maturity, a process known as “pod filling”. Five distinct stages in peanut pod development have been identified: stage S1 (R4) - pods with tiny embryos, stage S2 (R5) - seed growth, stage S3 (R6) - fully expanded but immature seeds, stage S4 (R7) - expanded and fully mature wet seeds, and stage S5 (R8) - mature dry seeds suitable for commercial use (25). Storage proteins start to accumulate as early as the S1 stage, while later in seed development, oil and fats, along with other storage nutrients, accumulate (26, 27). Consequently, during the early stages of seed maturation, when fats and fat globules are relatively scarce, the weight per volume concentration of peanut proteins, including major allergens, is relatively high.

The allergenicity of storage proteins in seeds may be influenced by their unique state during developmental stages. However, no studies have investigated the allergenicity of peanut maturational stages in children with peanut allergy. We have recently shown that prick-prick skin tests using a proprietary lyophilized powder from peanuts at stage S2 was capable of predicting peanut allergy in children with a positive predictive value of over 92%, serving as a far better predictor for the presence of peanut allergy compared to commercially approved diagnostics (28). Additionally, this same preparation was recently evaluated in an oral immunotherapy protocol and enabled the introduction of dietary peanut in highly allergic individuals with an increased safety profile (29). Therefore, this study aimed to explore the changes in allergenicity and protein allergen composition throughout the course of peanut development.

## 2 Materials and methods

### 2.1 Plant material and seed preparation

A late-maturing peanut variety with large pods and seeds, an indeterminate growth habit, and a relatively long period of seed development was used for the identification and production of sequential maturational stages of peanut seeds described as follows:

S1. Beginning seed: Initial seed growth occurs and seeds occupy less than 20% of the total pod volume (when the fruit is cut in cross-section).
S2. Developing seed: Early seed expansion takes place and seeds occupy approximately 50% of the total volume.
S3. Full seed: Pod's cavity is apparently filled by the seeds when fresh, but the pod is still not ready for harvest, as the pericarp is not yet colorized.
S4. Beginning maturity: Pod/seed shows natural coloration or blotching of inner pericarp or testa, respectively.
S5. Fully mature seed: Plants were grown to full maturation, defined by 65%–75% of all pods fully matured and dry.

Approximately 60 plants were grown in a net-house on beds 1.9 m wide. Seed production was carried out by manually harvesting the plants at the reproductive stage of beginning of maturity, stage S4 (R7), when at least one pod is matured (25). Since peanut is an indeterminate plant, seeds from each developmental stage (S1–S4) can be found during this particular stage. In addition, 15 plants were grown to full maturity, from which the seeds at stage S5 (R8) were extracted. After washing the pods to remove sand residues, pods were cut in cross-section with a razor blade and the seeds were extracted and sorted into five groups based on their developmental stage.

Developmental stages were assessed by two separate people (authors RH and YL) and a comparison was made between them to ensure that the definition of each stage is uniform. Seeds from stages S1–S5 were collected from all growing plants to ensure equal sampling. Within each developmental stage, seeds were divided into three biological replicates, immediately flash-frozen, and stored at −20°C until needed.

### 2.2 RNA expression of peanut allergen gene families

Changes in gene expression of allergenic proteins Ara h 1, Ara h 2, Ara h 3, and Ara h 6 throughout seed development were investigated. Sequences of these allergen genes were obtained from the peanut genome, available at peanutbase.org, using both the local tblastx in the transcript assembly and the KEGG database. To measure the expression levels of these genes, RNA-Seq libraries were constructed for each developmental stage, as previously described (27). The transcript quantification (i.e., number of reads per gene) was done by mapping the reads to the tetraploid peanut reference genome, also available on peanutbase.org, using the bowtie2 aligner (30) and the Expectation-Maximization method (RSEM), which estimates the maximum likelihood expression levels by handling the read mapping of uncertainty with a statistical model. To determine the expression of each allergenic gene family, the Reads Per Kilobase per Million (RPKM) values were summed for all homologous genes within each family. Sequences for Ara h 1, Ara h 2, Ara h 3, and Ara h 6 gene families were identified from the peanut 4X tetraploid genome assembly (PeanutBase.org) by using local tblastx in transcript assembly and the KEGG database with blast2go. Subsequently, the expression of all genes in each family were normalized to RPKM values for each developmental stage. Then, the values for each allergen family were added to obtain a single specific value. The relative expression ratio of the Ara h 2 gene family was calculated by separating total expression values into subgroups corresponding to either the A or B sub-genome, followed by dividing the Ara h 2 A values by the Ara h 2 B values.

### 2.3 Protein extraction

Following harvest, whole peanuts of each reproductive stage were lyophilized and ground into a dry powder. In addition, a mature seed sample (stage S5) was homogenized, and samples of all five stages were suspended in n-hexane for 2 rounds, passing the solution through a vacuum filter between rounds. The resulting defatted peanut flours were processed into whole protein extracts by dissolving 100 mg of flour in 1.5 ml of 20 mm Tris buffer (pH 8.5). Samples were vortexed for 30 s before rotating overnight at 4°C. Following 3 rounds of centrifugation for 5 min at 12,600 g, the supernatant was collected as the protein extract. Extract concentrations were determined by Bradford assay using known concentrations of bovine serum albumin (BSA; Sigma-Aldrich, Oakville, Canada) to construct a standard curve.

### 2.4 Relative quantification of specific peanut allergens

Relative levels of allergens Ara h 1 and Ara h 2 were quantified using the enzyme-linked immunosorbent assay (ELISA). Polystyrene 96-well microplates were coated overnight at 4°C with three replicates of four 100-fold serial dilutions of each protein extract (0.0001–100 µg/ml). Following blocking with 1% BSA (1 h RT), rabbit anti-Ara h 1 or Ara h 2 polyclonal antibody (1:1,000, 2 h RT; PA-AH1, PA-AH2, InBio, VA) was used as the primary antibody. Bound antibodies were visualized using horseradish peroxidase (HRP)-conjugated donkey anti-rabbit IgG antibody (1:1,000, 1 h RT; # 406401, BioLegend, CA). After incubation with 3,3′,5,5′-Tetramethylbenzidine (TMB) substrate (BioLegend), optical density (OD) values were measured at 450 nm with reference at 570 nm. All values were averaged over two technical replicates.

Relative levels of allergens Ara h 3 and Ara h 6 were quantified using commercial ELISA kits containing a plate pre-coated with anti-Ara h 3 monoclonal antibody 1E8 (EPC-AH3-1, InBio) or with anti-Ara h 6 monoclonal antibody 3B8 (EPC-AH6-1, InBio). Two replicates of four 10-fold serial dilutions of each protein extract (1–1,000 ng/ml) were added to the plate and incubated for 1 h at RT. Detection was achieved by adding biotinylated monoclonal antibody mixed with streptavidin-peroxidase provided by the kit. After incubation with TMB substrate, OD values were measured at 450 nm. All values were averaged over two technical replicates.

### 2.5 Determination of specific IgE responses

The specific IgE binding capacity of peanut proteins was analyzed using a similar ELISA protocol as described above. Pooled sera from 4 patients with high levels of peanut-specific IgE (median IgE for peanut: 474 kU/L, median age: 15 years old, 75% male) was diluted 1:250 in 1% BSA and used as the primary antibody (2 h RT). Biotinylated, goat anti-human IgE antibody (1:20,000, 1 h RT; #A80-108B, Bethyl Laboratories Inc., TX) followed by incubation with HRP-streptavidin (1:3,000, 1 h RT; BioLegend) were used for detection. To construct a standard curve, wells were coated with anti-human IgE capture antibody (1:1,000; #A80-108A, Bethyl Laboratories Inc.) and subsequently incubated with 10-fold serial dilutions of recombinant human IgE antibody starting at 100 ng/ml (ELISA Ready-SET-Go! Kit, #88-50610-77, Thermo Fisher Scientific).

### 2.6 Immunohistochemical staining

Ara h 2-specific staining was performed on seed sections taken from S2 and S4 developmental stages. The immunostaining process followed a protocol reported by Tsuda & Chuck with modifications (31). To prepare the tissue, the seeds were fixed in formalin, acetic acid, and ethanol (FAA) in a 5:5:90 ratio, gradually dehydrated in ethanol, fixed in paraffin, and dissected by microtome to obtain 10 µm-thick slices. For immunolocalization analysis, the fixed samples were initially incubated with 1% BSA for 1 h RT for blocking and then washed in PBST (1X PBS + 0.5% Tween20). The samples were then incubated with chicken anti-Ara h 2 [provided by Dr. Soheila Maleki (19)] as the primary antibody (1:100) and rabbit anti-chicken IgY (IgG, H + L, conjugated with Alexa Fluor-488; 303-545-003 Jackson Immunoresearch Laboratories) as the secondary antibody (1:200). The developing signal was detected by the Leica DNLB microscope and documented using a Nicon ds-fi 1 camera. For counterstaining, propidium iodide (red color) and calcofluor white (blue color) were used to stain the nucleus and cell wall, respectively.

### 2.7 Clinical study population

Children aged 1–18 years old with challenge-proven peanut allergy at the pediatric allergy clinic of the Safra Children's Hospital were evaluated between January 2017 and July 2019. Prior to study entry, parents and guardians received thorough counseling on the potential risks and signed informed consent forms approved by the Institutional Review Board (IRB) of the Sheba Medical Center and the national IRB, as required for any study involving children in Israel. After informed consent was obtained, all children underwent a full diagnostic workup, including a standardized open oral food challenge (OFC) to confirm the presence of peanut allergy.

### 2.8 Skin prick tests (SPTs)

SPTs were conducted on the forearms of children using single-head lancets. A positive control with histamine (1 mg/ml) and a negative control with glycerinated saline were used. In addition, commercial whole peanut extract (CPE; ALK-Abelló, Denmark) and lyophilized peanut powders from S1, S2, and S4 stages were used. After 15 min, the widest diameter of wheal and flare were measured. To avoid any interference, patients were instructed to abstain from using antihistamine-containing medications for at least one week before the procedure.

### 2.9 Statistical analyses

Statistical and data analyses were performed using SPSS (version 25, 2020, SPSS Inc., Chicago, IL, USA) and RStudio software (R version 4.2.2; v2022.07.2 + 576 Spotted Wakerobin Release, Boston, MA). Gene expression and ELISA values were expressed as mean ± standard error (SE) of 3 replicates. Analysis of Variance (ANOVA) and Tukey tests were used for multiple comparisons between OD and IgE binding values by ELISA. Statistical analyses of ELISA data were performed for an extract concentration of 1 µg/ml, within the dynamic range of samples of each condition. Repeated measures ANOVA was used for the comparison of SPT wheal sizes across the various developmental stages. A *p*-value of <0.05 was considered statistically significant in all cases.

## 3 Results

### 3.1 Molecular & genetic analysis of peanut seeds throughout development

**Figure 1:**
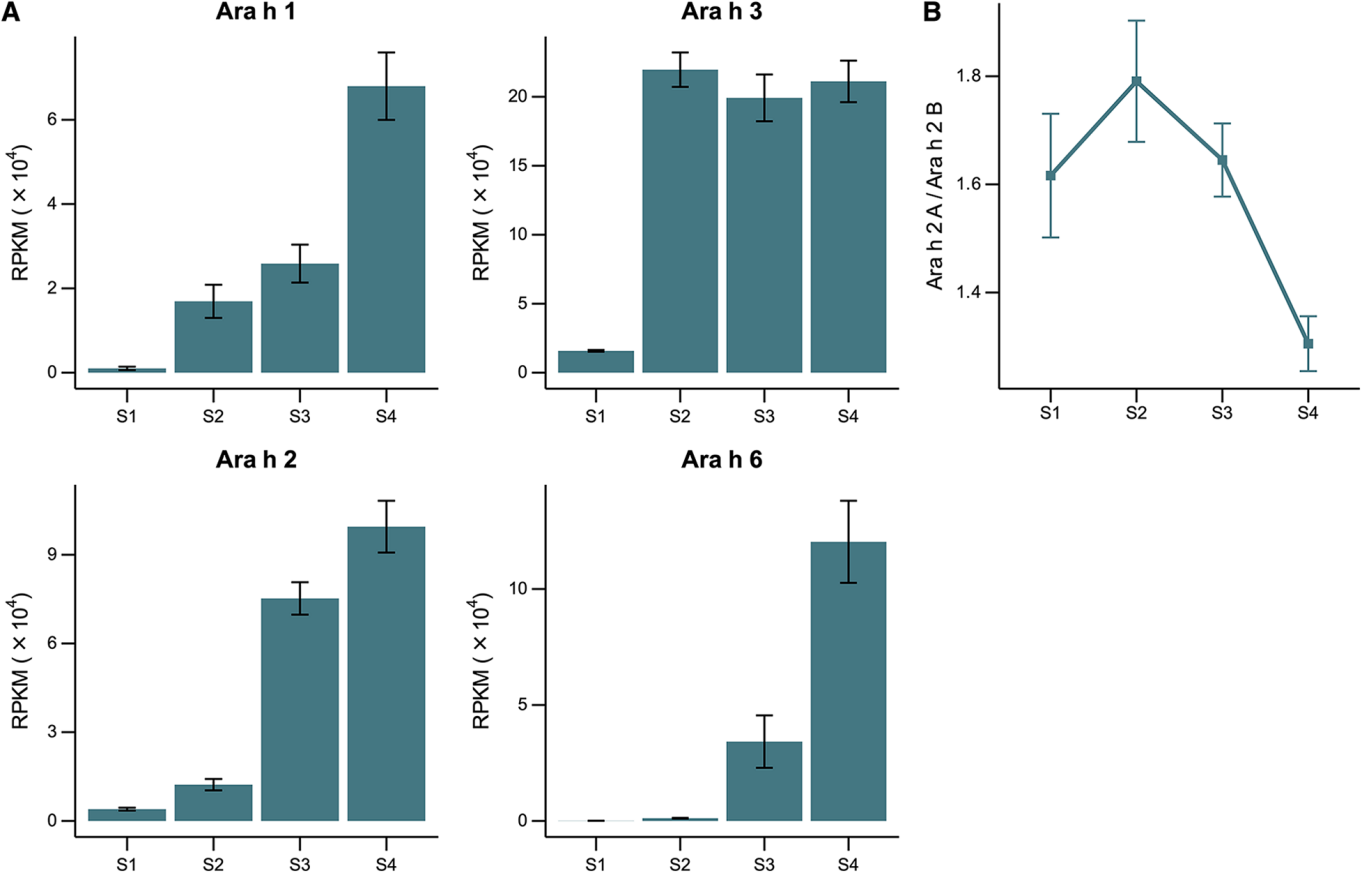
RNA expression throughout peanut seed development. **(A)** Expression levels of gene families encoding allergenic proteins Ara h 1, Ara h 2, Ara h 3, and Ara h 6 across the stages of seed development (S1–S4) represented as Reads Per Kilobase per Million (RPKM). **(B)** Relative genome expression of the homoeologous genes encoding Ara h 2 in each stage of development given as the ratio between the A- and B-Ara h 2 sub-genome expression within the polyploid peanut genome.

Expression levels of genes that encode peanut allergens Ara h 1, Ara h 2, Ara h 3, and Ara h 6 during seed development are presented in Figure 1A. The gene expression of all evaluated allergenic proteins was detectable at the earliest stage (S1) and increased throughout development. RNA levels of Ara h 3 increased more rapidly than the others, reaching its maximum at the S2 stage.

The Ara h 2 protein is encoded by 2 copies of homoeologous genes, one from the A-genome and the other from the B-genome of the AB allopolyploid peanut. Homoeologs are pairs of genes that originated by speciation and were brought back together in the same genome by allopolyploidization [i.e., multiple genomes duplicated via polyploidy in a single nucleus (32)]. Previous research has suggested that the B-genome homoeolog may be more allergenic than the A-genome homoeolog because of a 12-amino acid insertion of a hypersesnsitve epitope (33). The relative expression levels of the specific copies of the Ara h 2 gene were measured and we found a significant bias towards the less allergenic A-genome homoeolog in the early stages of seed development, particularly in stage S2 (Figure 1B). No significant differences in the expression bias of the other three gene families were observed (data not shown).

### 3.2 Relative allergen quantification and peanut-specific IgE binding

**Figure 2:**
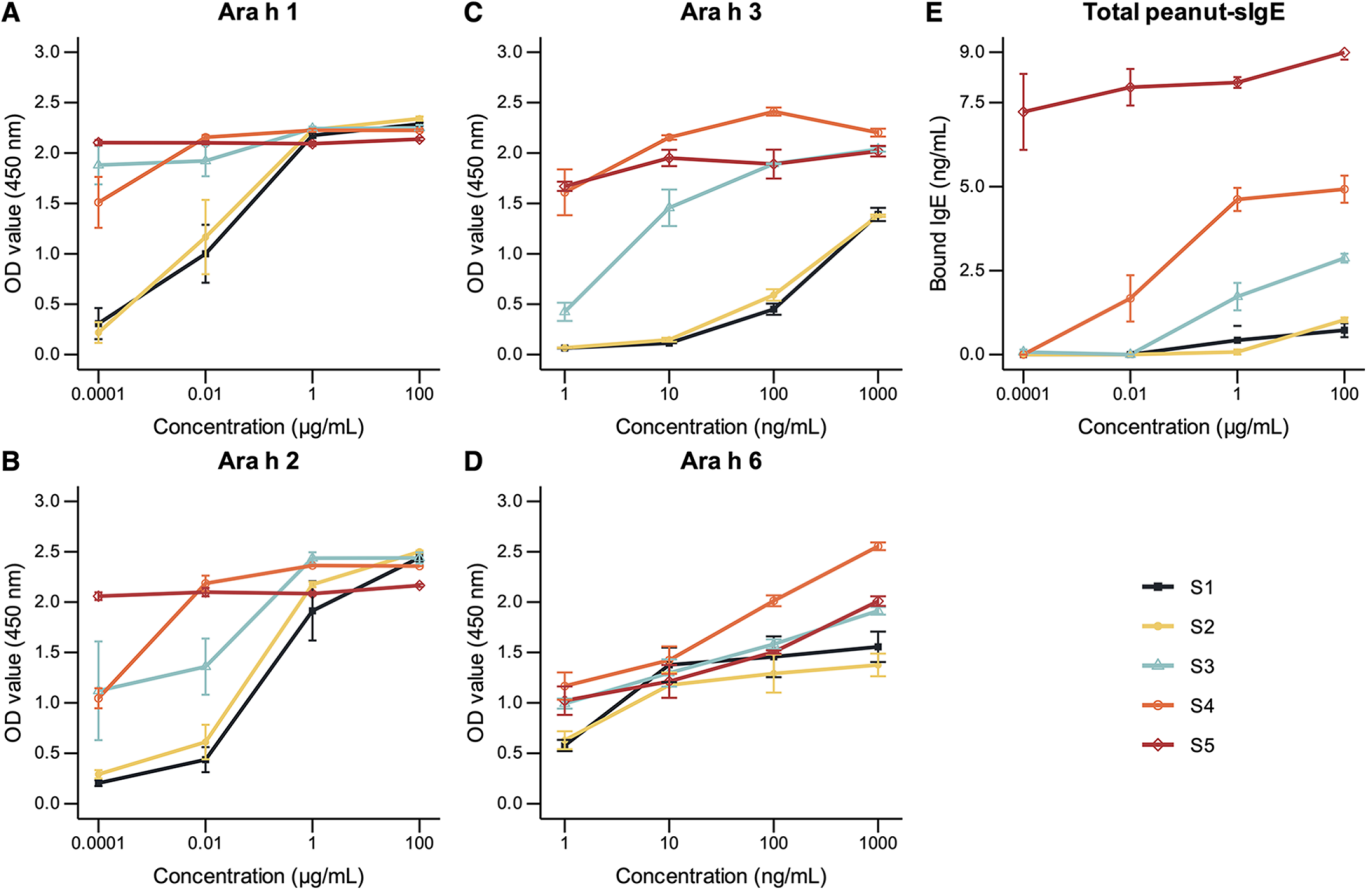
**(A–D)** relative quantification of Ara h 1, Ara h 2, Ara h 3, and Ara h 6 in peanuts of developmental stages S1, S2, S3, S4, and S5 by ELISA using allergen-specific antibodies. Plates were coated with a maximum concentration of 100 µg/ml of each developmental stage **(A,B)**. Plates were pre-coated with monoclonal antibody specific for Ara h 3 or Ara h 6, followed by the incubation of extracts of each developmental stage at a maximum concentration of 1,000 ng/ml **(C,D)**. **(E)** Total peanut-specific IgE quantification by ELISA using a pooled serum of 4 highly allergic subjects. Plates were coated with a maximum concentration of 100 µg/ml of each developmental stage. Optical density (OD) values were measured at 450 nm and referenced at 570 nm. Peanut-specific IgE levels were converted to concentration values using a standard curve constructed using known concentrations of human recombinant IgE. Error bars represent standard errors (*n* = 3) or minimum–maximum for Ara h 3 and Ara h 6 ELISAs (*n* = 2).

In Figures 2A–D, ELISA experiments were performed to quantify the relative amount of Ara h 1, Ara h 2, Ara h 3, and Ara h 6 present in whole protein extracts. The results showed that the detection levels of Ara h 1, Ara h 2, and Ara h 3 increased with maturity, demonstrating relatively low levels of detection in the earlier stages and the highest level of detection in the S5 stage. Minor differences in relative Ara h 6 levels were observed across the different stages, likely due to very low concentrations of the allergen in peanuts.

Peanut-specific IgE binding to peanut proteins was quantified using a pooled serum of 4 patients with high specific IgE to peanuts via the ELISA assay (Figure 2E). The results demonstrated an increase in IgE binding with peanut maturation, with the highest degree of binding to the mature, S5 stage peanut. This may suggest decreased allergenicity at earlier stages of peanut development.

### 3.3 Specific immunohistochemical staining of Ara h 2 proteins in seeds

**Figure 3:**
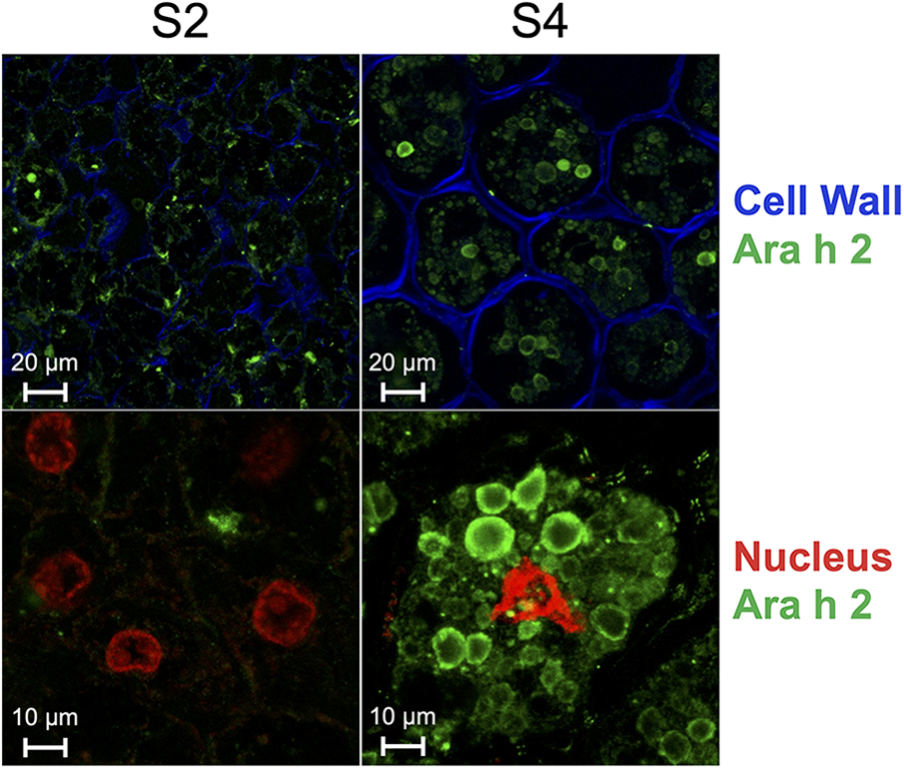
Immunolocalization of 12 μm-thick peanut seed sections of stages S2 and S4 using a primary chicken antibody specific for Ara h 2 and a secondary rabbit anti-chicken antibody coupled to Alexa fluor-488 (green). Cell wall was stained with calcofluor white (blue) and nucleus was stained with propidium iodide (red). Scale of top panels: 20 μm; scale of bottom panels: 10 μm.

Given that Ara h 2 has been established as a potent peanut allergen, we conducted a cellular-level analysis to gain further insights. Specifically, we examined anatomical sections of peanuts in the S2 and S4 stages to trace the development and formation of peanut protein bodies (Figure 3). Our findings indicate that Ara h 2 protein content is lower in S2 compared to S4. Additionally, we observed that Ara h 2 is more widely distributed within the cell in stage S2 and accumulates less within the tightly organized protein bodies than in S4.

### 3.4 Skin test responses to immature peanut seeds vs. commercial extracts

Table 1 displays the demographics and clinical characteristics of 60 peanut-allergic children enrolled in the study, of which 67% were male and 85% were under six years of age (interquartile range: 2.6–5.4). The children included were highly atopic, with many suffering from additional allergic comorbidities such as atopic dermatitis (50%), asthma (40%), allergic rhinitis (10%), and other food allergies (50%). The mean maximum amount of peanut protein that could be tolerated during oral food challenges before symptoms appeared was 168 mg [95% confidence interval (CI) 106–230 mg].

**Table 1:** Demographics and clinical characteristics of children enrolled for skin prick testing.

Characteristic	*N* = 60 (%) or mean (95% CI)
Age (years)	3.5 (2.6–5.4)^a^
Sex: male	40 (67%)
Atopic dermatitis	30 (50%)
Asthma	24 (40%)
Allergic rhinitis	6 (10%)
Other food allergy	30 (50%)
Maximum tolerated peanut dose upon OFC (mg)	168 (106, 230)
Skin prick test wheal diameter (mm)	
Stage S1	2.1 (1.7, 2.6)
Stage S2	7.5 (6.6, 8.4)
Stage S4	11.4 (10.2, 12.6)
Commercial peanut extract	10.4 (9.3, 11.6)

CI, confidence interval; OFC, oral food challenge.

^a^ Values for age are represented by median (interquartile range).

**Figure 4:**
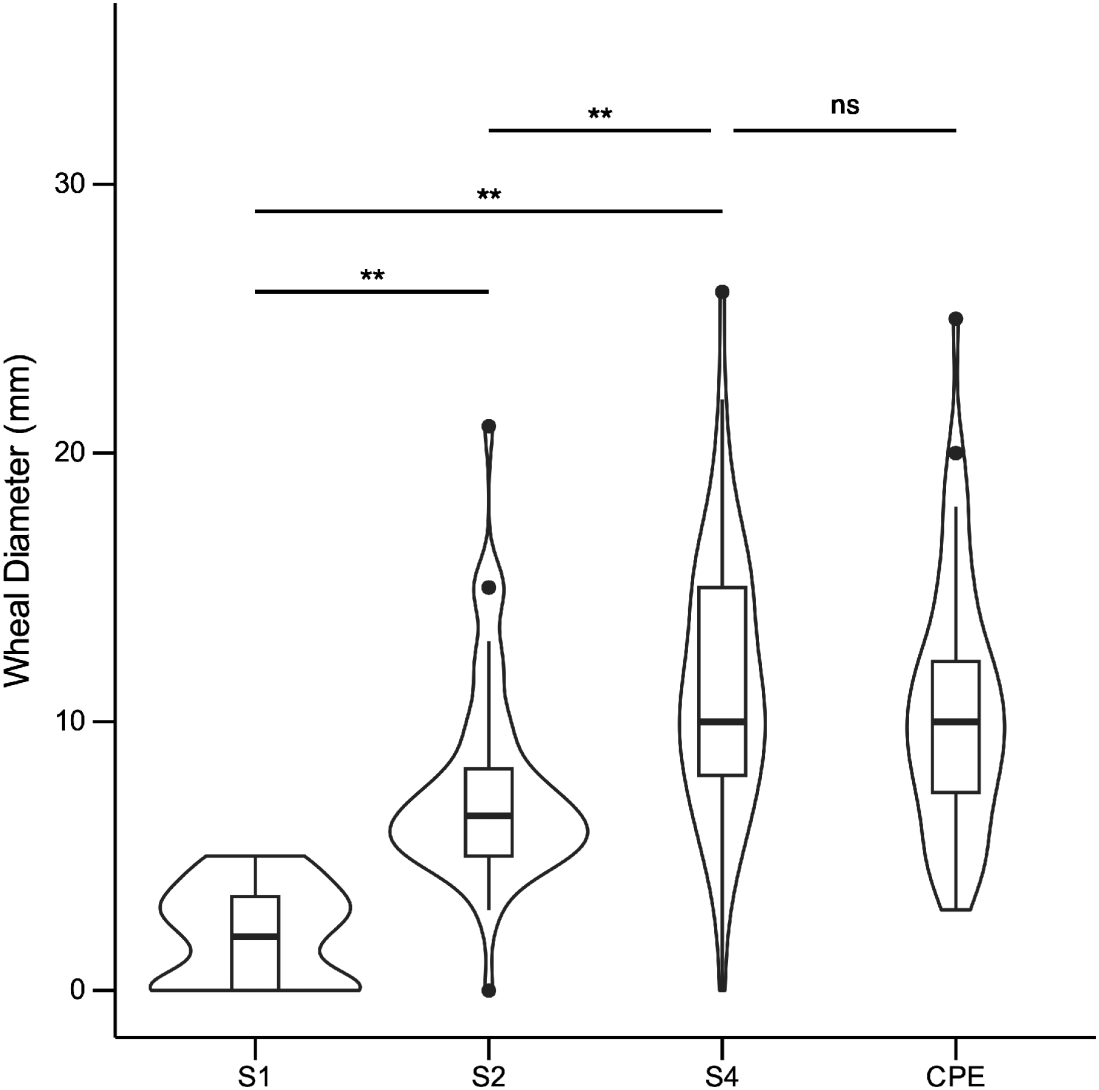
Skin test responses to seed reproductive stages S1, S2, S4, and to commercial peanut extract (CPE; ALK-abello) in 60 peanut allergic children. Values are reported as wheal diameters in millimeters (mm). Repeated measures ANOVA followed by pairwise Student's *t*-tests (Bonferroni correction) were performed to assess group differences. **: *p* < 0.001; ns, not significant (*p* ≥ 0.05).

Mean skin wheal diameters for immature peanut stages S1, S2, and S4 in comparison to the commercial peanut extract (CPE) are presented in Figure 4. Initially, the S3 stage was also used, but the results were highly similar to S2, and thus S3 was excluded for the remainder of the experiment to minimize patient discomfort. When using the S1 peanut, the mean wheal diameter was 2.1 mm (95% CI 1.7–2.6), while for S2, it was 7.5 mm (95% CI 6.6–8.4); for S4, it was 11.4 mm (95% CI 10.2–12.6), and for the CPE, it was 10.4 mm (95% CI 9.3–11.6). Repeated measures ANOVA showed significant differences between mean SPT wheal diameters to peanut developmental stages S2 and S4 with mean difference of 3.9 mm (*p *< 0.001), and between S1 and S4 with mean difference of 5.7 mm (*p* < 0.001).

## 4 Discussion

This study aimed to investigate the maturational changes in the allergenicity of peanuts throughout sequential developmental stages, including relative allergen levels, specific IgE binding, and allergen accumulation across stages. We observed lower expression levels of genes encoding major peanut allergens, decreased relative allergen levels, and reduced specific IgE binding levels to pooled sera in earlier developmental stages. We also observed a significant bias at earlier stages for the less allergenic A-genome homoeolog of Ara h 2, the peanut allergen most closely linked to anaphylaxis (34). Moreover, total Ara h 2 content was lower, was more widely distributed in peanut cells, and accumulated less in protein bodies in stage S2 than in S4.

The *in vivo* measurement of specific IgE-binding of immature peanut seeds, SPT wheal size, was significantly lower than that of mature peanuts in children with challenge-proven peanut allergy. Moreover, the allergenicity of peanuts appears to increase with peanut seed maturation. This study is the first to systematically explore the sequential development of allergenicity in immature peanut seeds.

The reduced allergenicity of immature peanuts can be explained by several mechanisms. Firstly, while total protein concentrations were similar, the relative expression and protein detection levels of the most potent allergenic proteins, Ara h 1, Ara h 2, and Ara h 3, were significantly lower in early seed developmental stages. This finding is supported by reduced IgE-binding to protein extracts of early peanut stages when compared to mature peanuts. Although detected Ara h 6 protein levels were similar across the range of concentrations evaluated, we believe this is partly due to the low abundance of Ara h 6 in peanuts at 5.5% (35) and it is possible a difference would be more evident at lower protein concentrations.

Secondly, the RNA and protein expression of Ara h 2 in the S1 and S2 stages is biased towards the production of its less allergenic isoform, rendering these seeds naturally hypoallergenic. Changes in expression bias between homoeologous genes in allopolyploid plants is a well-known phenomenon and can have significant evolutional outcomes, such as alternate or novel functions (36). This phenomenon of homoeolog-specific gene expressions can vary vastly between organs, seed development stages, and even in the same tissue due to environmental stresses (27, 37, 38). Therefore, it is not surprising that changes in the relative expression between the two Ara h 2 homoeologs occur during seed development, even between the S4 and S5 stages, which are two forms of mature peanuts (i.e., before and after pod drying).

Another possible explanation for the reduced specific IgE-binding of immature seeds could be the organization and composition of the protein bodies, the cellular organelles responsible for the long-term storage and accumulation of proteins within peanut cells. In the S2 maturational stage, these protein bodies are smaller and highly dispersed throughout the cell. Additionally, immature seeds have relatively higher water contents and carbohydrate levels, and lower oil and fat levels, which may render the allergenic proteins more amenable to digestion or to thermally induced denaturation. Decreases in peanut-specific IgE-binding of immature peanut seeds may not only be a result of decreased amounts of allergenic proteins, but also of profound changes in the composition, conformation, glycosylation, and/or conglomeration of the antigens presented to the immune system. These are relevant topics for further research.

It is important to note that while the use of an immature peanut with reduced allergenic potential may lead to fewer adverse reactions upon consumption, further studies are necessary to evaluate the risk of initial sensitization to peanut and the ability of immature peanuts to induce desensitization via OIT.

## 5 Conclusions

The observed decreases in IgE-binding and wheal sizes measured by SPT of immature peanut seeds may be a result of both decreased amounts of allergenic proteins, as well as of significant changes in the composition and/or conglomeration of the peanut allergens interacting with the immune system. Using these naturally hypoallergenic peanuts may enable novel, safer pathways for the treatment of life-threatening peanut allergy.

## Data Availability

The datasets presented in this study can be found in online repositories. The names of the repository/repositories and accession number(s) can be found below: https://www.ncbi.nlm.nih.gov/, PRJNA982443.

## References

[1] Renz H Allen KJ Sicherer SH Sampson HA Lack G Beyer K Food allergy. Nat Rev Dis Primers. (2018) 4:17098. 10.1038/nrdp.2017.98 29300005

[2] Sicherer SH Sampson HA. Food allergy: a review and update on epidemiology, pathogenesis, diagnosis, prevention, and management. J Allergy Clin Immunol. (2018) 141(1):41–58. 10.1016/j.jaci.2017.11.003 29157945

[3] Clarke AE Elliott SJ St. Pierre Y Soller L La Vieille S Ben-Shoshan M. Temporal trends in prevalence of food allergy in Canada. J Allergy Clin Immunol Pract. (2020) 8(4):1428–30. 10.1016/j.jaip.2019.10.021 31706046

[4] Sicherer SH Muñoz-Furlong A Godbold JH Sampson HA. US prevalence of self-reported peanut, tree nut, and sesame allergy: 11-year follow-up. J Allergy Clin Immunol. (2010) 125(6):1322–6. 10.1016/j.jaci.2010.03.029 20462634

[5] Warren C Lei D Sicherer S Schleimer R Gupta R. Prevalence and characteristics of peanut allergy in US adults. J Allergy Clin Immunol. (2021) 147(6):2263–70. 10.1016/j.jaci.2020.11.046 33579526 PMC12341317

[6] Sampson HA Bernstein D Aceves S Bock SA James J Jones S Food allergy: a practice parameter update-2014. J Allergy Clin Immunol. (2014) 134(5):1016–25. 10.1016/j.jaci.2014.05.013 25174862

[7] Jung M Jeong HI Kyung Y Kim SK Lee JS Choi M Natural course and prognostic factors of immediate-type peanut allergy in children. Int Arch Allergy Immunol. (2021) 182(11):1072–6. 10.1159/000516811 34419947

[8] Bird JA Spergel JM Jones SM Rachid R Assa'ad AH Wang J Efficacy and safety of AR101 in oral immunotherapy for peanut allergy: results of ARC001, a randomized, double-blind, placebo-controlled phase 2 clinical trial. J Allergy Clin Immunol Pract. (2018) 6(2):476–85.e3. 10.1016/j.jaip.2017.09.016 29092786

[9] Vickery BP Vereda A Casale TB Beyer K du Toit G Hourihane JO AR101 Oral immunotherapy for peanut allergy. N Engl J Med. (2018) 379(21):1991–2001. 10.1056/NEJMoa1812856 30449234

[10] Cummings AJ Knibb RC King RM Lucas JS. The psychosocial impact of food allergy and food hypersensitivity in children, adolescents and their families: a review. Allergy. (2010) 65(8):933–45. 10.1111/j.1398-9995.2010.02342.x 20180792

[11] Becker WM Jappe U. Peanut allergens. Chem Immunol Allergy. (2014) 100:256–67. 10.1159/000359916 24925406

[12] Mueller GA Maleki SJ Pedersen LC. The molecular basis of peanut allergy. Curr Allergy Asthma Rep. (2014) 14(5):429. 10.1007/s11882-014-0429-5 24633613 PMC4785306

[13] Skypala IJ Asero R Barber D Cecchi L Diaz Perales A Hoffmann-Sommergruber K Non-specific lipid-transfer proteins: allergen structure and function, cross-reactivity, sensitization, and epidemiology. Clin Transl Allergy. (2021) 11(3):e12010. 10.1002/clt2.12010 34025983 PMC8129635

[14] Calamelli E Liotti L Beghetti I Piccinno V Serra L Bottau P. Component-resolved diagnosis in food allergies. Medicina (Kaunas). (2019) 55(8):498. 10.3390/medicina55080498 31426616 PMC6723663

[15] Asarnoj A Nilsson C Lidholm J Glaumann S Östblom E Hedlin G Peanut component Ara h 8 sensitization and tolerance to peanut. J Allergy Clin Immunol. (2012) 130(2):468–72. 10.1016/j.jaci.2012.05.019 22738678

[16] Foo ACY Mueller GA. Abundance and stability as common properties of allergens. Front Allergy. (2021) 2:769728. 10.3389/falgy.2021.769728 35386965 PMC8974735

[17] Vissers YM Iwan M Adel-Patient K Stahl Skov P Rigby NM Johnson PE Effect of roasting on the allergenicity of major peanut allergens Ara h 1 and Ara h 2/6: the necessity of degranulation assays. Clin Exp Allergy. (2011) 41(11):1631–42. 10.1111/j.1365-2222.2011.03830.x 21801247

[18] Moghaddam AE Hillson WR Noti M Gartlan KH Johnson S Thomas B Dry roasting enhances peanut-induced allergic sensitization across mucosal and cutaneous routes in mice. J Allergy Clin Immunol. (2014) 134(6):1453–6. 10.1016/j.jaci.2014.07.032 25253515 PMC4861634

[19] Comstock SS Maleki SJ Teuber SS. Boiling and frying peanuts decreases soluble peanut (*Arachis Hypogaea*) allergens Ara h 1 and Ara h 2 but does not generate hypoallergenic peanuts. PLoS One. (2016) 11(6):e0157849. 10.1371/journal.pone.0157849 27310538 PMC4911009

[20] Turner PJ Mehr S Sayers R Wong M Shamji MH Campbell DE Loss of allergenic proteins during boiling explains tolerance to boiled peanut in peanut allergy. J Allergy Clin Immunol. (2014) 134(3):751–3. 10.1016/j.jaci.2014.06.016 25065723

[21] Young CT Pattee HE Schadel WE Sanders TH. Microstructure of peanut (*Arachis hypogaea* L. cv. “NC 7”) cotyledons during development. LWT Food Sci Technol. (2004) 37(4):439–45. 10.1016/j.lwt.2003.10.016

[22] Palomares O Akdis M Martin-Fontecha M Akdis CA. Mechanisms of immune regulation in allergic diseases: the role of regulatory T and B cells. Immunol Rev. (2017) 278(1):219–36. 10.1111/imr.12555 28658547

[23] Shreffler WG Beyer K Chu T-HT Burks AW Sampson HA. Microarray immunoassay: association of clinical history, in vitro IgE function, and heterogeneity of allergenic peanut epitopes. J Allergy Clin Immunol. (2004) 113(4):776–82. 10.1016/j.jaci.2003.12.588 15100687

[24] Periasamy K Sampoornam C. The morphology and anatomy of ovule and fruit development in *Arachis hypogaea* L. Ann Bot. (1984) 53(3):399–411. 10.1093/oxfordjournals.aob.a086703

[25] Boote KJ. Growth stages of peanut (*Arachis hypogaea* L.). Peanut Sci. (1982) 9(1):35–40. 10.3146/i0095-3679-9-1-11

[26] Pickett TA. Composition of developing peanut seed. Plant Physiol. (1950) 25(2):210–24. 10.1104/pp.25.2.210 16654285 PMC437428

[27] Gupta K Kayam G Faigenboim-Doron A Clevenger J Ozias-Akins P Hovav R. Gene expression profiling during seed-filling process in peanut with emphasis on oil biosynthesis networks. Plant Sci. (2016) 248:116–27. 10.1016/j.plantsci.2016.04.014 27181953

[28] Kidon MI Yahia SH Machnes-Maayan D Levy Y Frizinsky S Maoz-Segal R Diagnosis of peanut allergy in preschool children: the impact of skin testing with a novel composition of peanuts. Front Pediatr. (2021) 9:739224. 10.3389/fped.2021.739224 34917557 PMC8670606

[29] Kidon MI Shavit R Levy Y Haj Yahia S Machnes-Maayan D Frizinsky S Peanut oral immunotherapy using an extensively heated and baked novel composition of peanuts. Pediatr Allergy Immunol. (2024) 35(5):e14146. 10.1111/pai.14146 38783409

[30] Langmead B Salzberg SL. Fast gapped-read alignment with bowtie 2. Nat Methods. (2012) 9(4):357–9. 10.1038/nmeth.1923 22388286 PMC3322381

[31] Tsuda K Chuck G. Heat induced epitope retrieval (HIER) assisted protein immunostaining in maize. Bio Protoc. (2019) 9(11):e3260. 10.21769/BioProtoc.3260

[32] Glover NM Redestig H Dessimoz C. Homoeologs: what are they and how do we infer them? Trends Plant Sci. (2016) 21(7):609–21. 10.1016/j.tplants.2016.02.005 27021699 PMC4920642

[33] Chatel J-M Bernard H Orson FM. Isolation and characterization of two complete Ara h 2 isoforms cDNA. Int Arch Allergy Appl Immunol. (2003) 131(1):14–8. 10.1159/000070429 12759484

[34] Hemmings OM Du Toit GF Radulovic SMD Lack GF Santos AFMDP. Ara h 2 is the dominant peanut allergen despite similarities with Ara h 6. J Allergy Clin Immunol. (2020) 146(3):621–30. 10.1016/j.jaci.2020.03.026 32298698 PMC7482438

[35] Marsh JT Palmer LK Koppelman SJ Johnson PE. Determination of allergen levels. Isoforms and their hydroxyproline modifications among peanut genotypes by mass spectrometry. Front Allergy. (2022) 3:872714. 10.3389/falgy.2022.872714 35769555 PMC9234871

[36] Hu G Grover CE Arick MA II Liu M Peterson DG Wendel JF. Homoeologous gene expression and co-expression network analyses and evolutionary inference in allopolyploids. Brief Bioinform. (2021) 22(2):1819–35. 10.1093/bib/bbaa035 32219306 PMC7986634

[37] Hovav R Faigenboim-Doron A Kadmon N Hu G Zhang X Gallagher JP A transcriptome profile for developing seed of polyploid cotton. Plant Genome. (2015) 8(1):eplantgenome2014.08.0041. 10.3835/plantgenome2014.08.0041 33228286

[38] Lee JS Adams KL. Global insights into duplicated gene expression and alternative splicing in polyploid *Brassica napus* under heat, cold, and drought stress. Plant Genome. (2020) 13(3):e20057. 10.1002/tpg2.20057 33043636 PMC12806937

